# Consumption of probiotic yogurt and vitamin D‐fortified yogurt increases fasting level of GLP‐1 in obese adults undergoing low‐calorie diet: A double‐blind randomized controlled trial

**DOI:** 10.1002/fsn3.2816

**Published:** 2022-07-27

**Authors:** Shima Hajipoor, Azita Hekmatdoost, Yahya Pasdar, Reza Mohammadi, Meysam Alipour, Mansour Rezaie, Seyed Mostafa Nachvak, Celso Fasura Balthazar, Mohammad Reza Sobhiyeh, Amir Mohammad Mortazavian, Adriano G. Cruz

**Affiliations:** ^1^ Student Research Committee Department of Nutritional Sciences School of Nutritional Sciences and Food Technology Kermanshah University of Medical Sciences Kermanshah Iran; ^2^ Department of Nutrition and Food Science Beheshti University of Medical Science Tehran Iran; ^3^ Department of Nutritional Sciences School of Nutritional Sciences and Food Technology Kermanshah University of Medical Sciences Kermanshah Iran; ^4^ Department of Food Science and Technology, School of Nutrition Sciences and Food Technology Kermanshah University of Medical Sciences Kermanshah Iran; ^5^ Department of Nutrition Shoushtar Faculty of Medical Sciences Shoushtar Iran; ^6^ Research Centre for Environmental Determinacies of Health Health Institute School of Public Health Kermanshah University of Medical Sciences Kermanshah Iran; ^7^ Department of Food Technology Veterinary College Federal Fluminense University Rio de Janeiro Brazil; ^8^ Vascular and Endovascular Surgeon Department of Surgery Imam Reza Hospital Kermanshah University of Medical Science Kermanshah Iran; ^9^ Department of Food Science and Technology National Nutrition and Food Technology Research Institute Faculty of Nutrition Sciences Food Science and Technology Shahid Beheshti University of Medical Sciences Tehran Iran; ^10^ Instituto Federal de Educação Ciência e Tecnologia do Rio de Janeiro (IFRJ) Departamento de Alimentos Rio de Janeiro Brazil

**Keywords:** ghrelin, GLP‐1, PY, PYY, satiety, vitamin D‐fortified yogurt

## Abstract

Energy restriction and manipulation of macronutrient composition of the diet are the main approaches that are used by people who aim to lose weight. When such strategies are employed, appetite and endocrine regulators of satiety, such as gut peptides, all are deeply affected. The gut microbiota–brain axis controls energy homeostasis in humans by affecting central satiety and gut peptides. The purpose of this study was to evaluate if the synergistic effect of probiotics and vitamin D in yogurt matrix can modulate this effect. In the double‐blind, randomized, placebo‐controlled trial, 140 obese adults were randomly allocated into four groups: 1) regular yogurt plus low‐calorie diet; 2) PY plus low‐calorie diet; 3) vitamin D‐fortified yogurt plus low‐calorie diet, and 4) probiotic and vitamin D co‐fortified yogurt plus low‐calorie diet. All groups were encouraged to increase their physical activity. Glucagon‐like peptide‐1 (GLP‐1), peptide Tyrosin‐Tysrosin (PYY), ghrelin, anthropometric variables, insulin, fasting blood sugar (FBS), insulin resistance/sensitivity, 1,25(OH)_2_ D_3_, dietary intake, and physical activity were measured before and after 10 weeks. The difference between groups for GLP‐1 after 10 weeks was significant after adjusting for baseline GLP‐1 and protein intake as confounders. PY showed the largest effect size (ES) on GLP‐1 (*p* = 14.2) and FBS (*p* = 14) compared with others. Pairwise comparison of yogurts effect sizes on GLP‐1 showed a significant difference in group 1 vs. group 2 (*p* = .001), group 1 vs. group 3 (*p* = .003), and group 1 vs. group 4 (*p* = .048). Vitamin D‐fortified yogurt had the largest effect size on the serum level of vitamin D and it showed a significant difference with RY (*p* = .018) and PY (*p* = .002). Consumption of vitamin D‐fortified yogurt and PY could be regarded as a promising approach during calorie restriction.

## Graphical abstract

This study assesses whether the synergistic effect of vitamin D and probiotic‐fortified yogurt on gut hormones affects satiety.

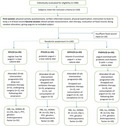

## 1 INTRODUCTION

According to the World Health Organization (WHO), obesity is characterized by an excess storage of fat arising from an imbalance between energy intake and energy expenditure (EE) (Blomain et al., 2013). It is well known that individuals with obesity are at a greater risk for numerous medical conditions (Pourshahidi, 2015), such as chronic and life‐threatening disorders like type 2 diabetes (Jafari‐Adli et al., 2014), cardiovascular disease, hyperlipidemia, and sleep apnea, which consequently impose a huge financial burden on the healthcare system (Bialo, 2015). This chronic disease is a multifaceted problem with many contributing factors including but not limited to genetics, overeating, and sedentary lifestyle.

Lifestyle modification including dietary interventions combined with exercise has always been the main therapeutic intervention for controlling obesity. However, substantial evidence shows that weight loss may be associated with biological adaptations including gradual change in the secretion of gut peptides which initiate an impaired cycle with progressively deteriorating appetite regulation which promotes overfeeding.

Glucagon‐like peptide‐1 and PYY are anorexigenic hormones which enhance satiety, probably through delay of gastric emptying. Ghrelin is the only peripheral orexigenic peptide that has been described. Previous studies have shown that the gut peptides are affected by clinical status in obese people. On the other hand, it is different from lean to obese individuals (Carlson et al., 2009; le Roux et al., 2006; Zwirska‐Korczala et al., 2007). There is overwhelming evidence showing that ghrelin secretion attenuates during diet‐induced obesity (DIO) (Bialo et al., 2015; Zigman et al., 2016). Furthermore, in DIO, ghrelin can no longer respond to food intake, which induces hyperphagia (Yang et al., 2009). Hypothalamic response regarding controlling food intake becomes resistant to ghrelin during obesity (English et al., 2002; Perreault, et al., 2004). In addition, the postprandial PYY and GLP‐1 response attenuate in obese people (le Roux et al., 2006; Madsbad, 2013). It has been proposed that there is an inverse relationship between postprandial GLP‐1 and insulin concentration. Consequently, insulin resistance might be promoted by further weight gain due to impaired GLP‐1 response.

There is accumulating evidence that alteration in the secretion of peripheral hormones is an adaptive response to negative energy balance. This adaptive response might underlie the common inclination for increasing hunger in obese people who have undergone diet programs (3). Several studies have indicated that modest diet‐induced weight loss imposes a long‐term and profound reduction in GLP‐1 and PYY (4,5) and increases ghrelin (1,8). This may at least in part explain why weight loss through caloric restriction is often too difficult to achieve and/or maintain for obese individuals (8,15,16). Central resistance to ghrelin might be related to insulin resistance (Chabot et al., 2014).

The role of gut–brain axis in controlling energy homeostasis has captured researcher's attention in the last decade. Experimental models highlight several mechanisms regarding the involvement of gut microbiota in host energy balance by influencing gut–brain axis (Neary et al., 2003). Manipulation of gut flora composition by delivering probiotic bacteria either as isolated bacteria or in food that has been fortified by probiotics can be regarded as an attractive treatment strategy for obesity management. The anorexigenic benefits of probiotics have been attributed to metabolites of these bacteria such as short‐chain fatty acids (SCFAs), which have been shown to mediate central satiety signaling pathways and peripheral hormones (Fetissov, 2016). In parallel, accumulating studies have addressed that vitamin D deficiency is more prevalent in obese people compared with normal population (Marcotorchino et al., 2014; Vimaleswaran et al., 2013). Furthermore, Vitamin D status is associated with the composition and function of the intestinal microbiome.

There is growing evidence suggesting the synergistic impact of combined vitamin D and probiotic administration on improving dysbiosis of gut microbiota in people with metabolic disorders (Jones et al., 2017; Ostadmohammadi et al., 2019). The basis of this approach relies on probiotics effect increasing vitamin D levels. In addition, probiotics might have synergistic effects with vitamin D through improving the expression of vitamin D receptors (Pannacciulli et al., 2006). Therefore, modulating the microbiota–gut–brain axis by probiotics plus improving vitamin D levels might provide a novel target to treat mental and metabolic disorders.

To the best of our knowledge, no study has addressed the synergist effect of probiotic and vitamin D during weight loss program on gut peptides up to now. Thus, the current study was designed to evaluate if probiotics and vitamin D can modulate gut peptides' changes during calorie restriction.

## 2 MATERIALS AND METHODS

### 2.1 Subjects

A total of 140 obese healthy adults (40 men and 100 women) were randomly assigned into four groups (35 subjects in each group). The subjects were selected between November and December 2017 and were recruited through advertising in public places on the basis of the following inclusion and exclusion criteria that were verified during telephone interviews. Inclusion criteria included the following: willingness to lose weight; body mass index (BMI) >30 kg/m^2^ without associated comorbidities (insulin‐dependent diabetes, chronic kidney disease, cancer); no immunocompromised conditions or anemia; absence of breastfeeding, pregnancy, or menopause (determined by the cessation of menstruation); no intake of vitamin D for 1 month prior to the study initiation; no antibiotic treatment for the last 1 month, and no intake of probiotic, prebiotic, and symbiotic supplement and/or probiotic, prebiotic, and symbiotic enriched products 1 month before the study initiation.

Exclusion criteria included the following: antibiotic therapy, intake of medication affecting satiety and vitamin D metabolism, body weight and/or energy expenditure, nonconsumption of more than 90% of yogurts regularly, not following the given diet, experiencing nausea, vomiting, diarrhea, previously having been diagnosed with hormone disorders including hypothyroid, hyperthyroid, polycystic ovary syndrome (PCOS), breast and uterus cyst, and additional factors that might interfere with the measurement of outcomes or with the success of the intervention (e.g., inability to attend for receiving yogurt regularly or diet therapy sessions).

### 2.2 Ethical approval

This study was performed between November 2018 and April 2019 in the Kermanshah University of Medical Sciences (KUMS) at the Faculty of Nutritional Science. The study proposal was approved by Human Research Ethics Committee at the KUMS (Approval No: KUMS.REC1395467). The clinical trial has been registered at IRCT with the registration number IRCT201608299856N3.

### 2.3 Study design

**FIGURE 1:**
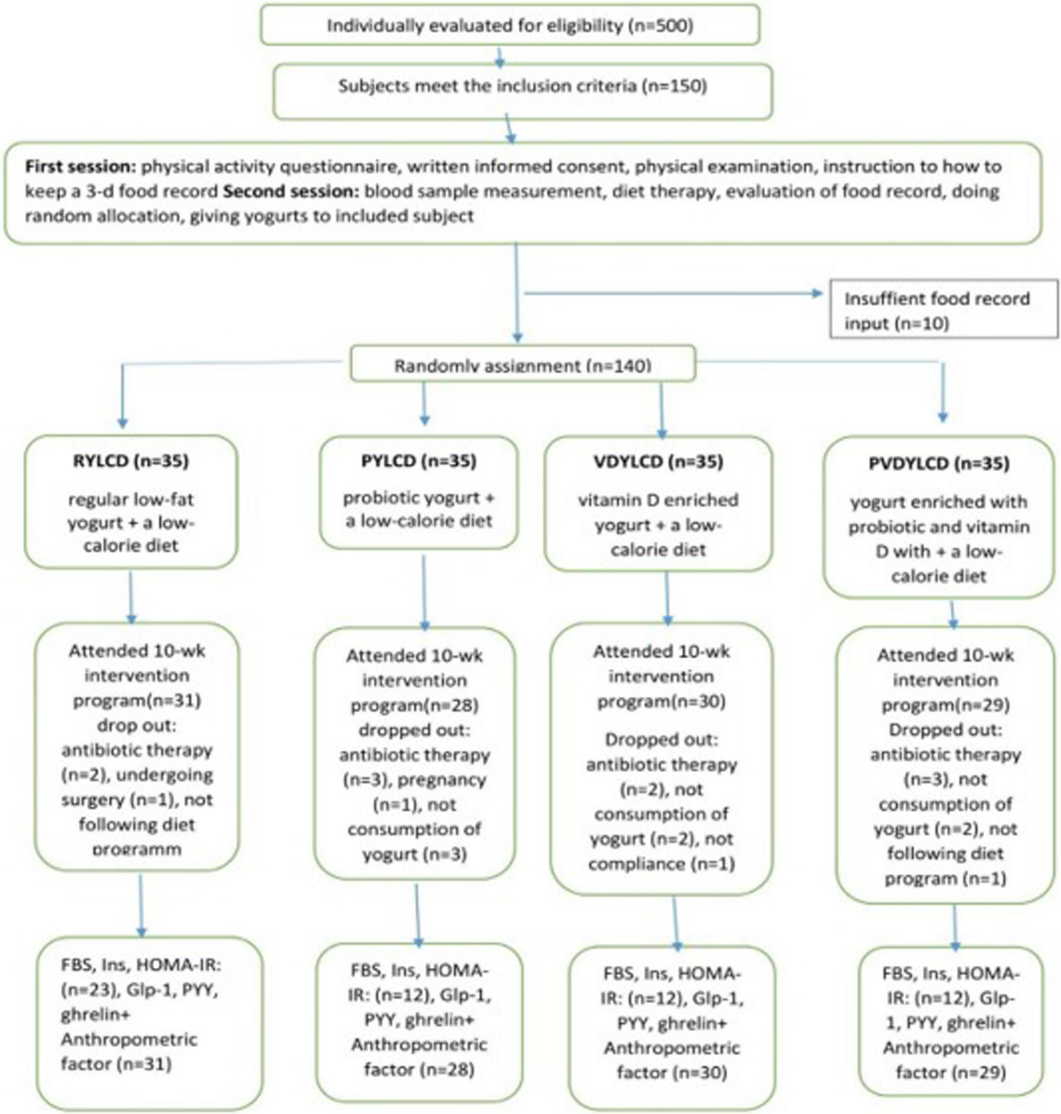
Study design of the effect of probiotic plus vitamin D fortified yogurt on the gut peptides

This randomized double‐blind controlled clinical trial was designed to examine whether probiotic and vitamin D‐enriched yogurt imposes any modulating effect on diet‐induced changes on gut peptides. The study period consisted of 10 weeks and the participants consumed 100 g of yogurt per day. Written informed consent was obtained from all the subjects. At baseline, participants were randomly assigned by computer‐generated random numbers to one of four intervention groups. Furthermore, participants were assigned to receive either: 1) regular low‐fat yogurt with a low‐calorie diet, 2) probiotic low‐fat yogurt with a low‐calorie diet, 3) vitamin D‐fortified low‐fat yogurt with a low‐calorie diet, and 4) low‐fat yogurt fortified with probiotics and vitamin D with a low‐calorie diet. The low‐fat yogurt had 0.5 g/100 g of fat. Random assignment was done by an independent researcher who was not involved in the data collection, analysis, or reporting performed. All subjects and the main investigator remained blinded until the analysis of results. Subjects were asked not to consume any probiotic and vitamin D‐containing food, yogurt, or its products during the study. Participants were asked to attend the laboratory every 10 days to get their yogurt. The participants’ diagram is depicted in Figure 1.

### 2.4 Interventions

The regular yogurt (RY) which contained only starter cultures of *Streptococcus thermophilus* and *Lactobacillus delbruecki* ssp. *bulgaricus* commonly has been used in conventional yogurt production (Mortazavian et al., 2006). PY (PY) was prepared with the starter cultures containing *Streptococcus thermophilus* and *Lactobacillus delbruecki* ssp. *bulgaricus*. Then, probiotic culture of two strains of Lactobacilli (*Lactobacillus acidophilus LA*‐*5*) and bifidobacteria (*Bifidobacterium* lactis BB‐12 cells) at levels of 4 × 10^7^ colony‐forming units (CFU)/g for each strain were added. This concentration was based on the minimum recommended consumption of probiotic cultures (>10^7^ CFU/g or 10^9^ per portion [100 g]). Vitamin D‐enriched yogurt was prepared by adding 1000 international unit (IU) vitamin D/100 g. The concentration of Vitamin D was based on the recommendation of the Institute of Medicine (2010) (600 IU/day), and on previous studies. There was no difference in composition, color, taste, and texture among all the yogurts.

Taking into consideration food preferences of participants, the diet program was designed based on the subject's food diary records in individual diet therapy sessions. The diet program was designed to introduce a 500–1000 kcal energy deficit to the estimated calorie needs based on their baseline ideal body weight (IBW).

Subjects were encouraged to gradually increase physical activity to achieve 45–60 min' moderate activity three times a week. The shortened form of the international physical activity questionnaire (IPAQ) was used to estimate the weekly energy expended in physical tasks as was represented by the metabolic equivalent of task (MET) score (Mousa et al., 2017).

### 2.5 Clinical assessments

Blood samples were taken after an overnight (at least 12 h fasting, at baseline, and after the 10‐weeks intervention. Serum was obtained by centrifugation (20 min) based on the kit protocol and stored at −40°C. FBS and insulin were serum evaluated. The homeostatic model of insulin resistance (HOMA‐IR) and quantitative insulin sensitivity check index (QUICKI) were calculated. The concentration of appetite‐related factors (Ghrelin, GLP‐1, and PYY) was measured with enzyme‐linked immunosorbent assay by using Human Ghrelin(Eastbiopharm; Cat. No: CK‐E10638), Human Glucagon‐like peptide‐1 (GLP‐1) (Eastabiopharm; Cat. No: CK‐e91926), and Human Peptide YY Elisa Kit (Eastabiopharm; Cat. No: CK‐e10370), respectively. Insulin was measured by using Monobind Insulin Kit (Monobind Inc, Product Code: 5825–300). FBS was measured with the enzymatic method by using an autoanalyzer machine (Cobas). Vitamin D was measured by using Monobind vitamin D kit. The HOMA‐IR index was calculated as HOMA‐IR =fasting serum glucose in milligrams per deciliter ×fasting insulin in micro units per milliliter/405. Insulin sensitivity was calculated according to quantitative insulin sensitivity check index (QUICKI =1/[log(I0) +log(G0)]).

### 2.6 Demographic assessments

Data about age, gender, education, job, and medical history were collected using a demographic questionnaire. Evaluation of sun exposure was based on self‐report.

### 2.7 Anthropometric measurements

Height was measured at the beginning of the study to the nearest 0.1 cm. Weight was measured by the digital calibrated scale (Seca) to the nearest 0.1 kg, while subjects wore light clothing and no shoes. BMI was calculated in kg/m^2^. Waist circumference (WC) and hip circumference (HC) were determined by using a rigid tape. The waist/hip ratio (WHR) was calculated. Furthermore, body composition including lean body mass (LBM), total body water (TBW), and fat mass (FM) was measured by using Body Analyzer, model Jawon plus avis.

### 2.8 Dietary intake assessments

Food and beverage intakes were assessed with the use of 3‐day food diary record. All subjects were instructed on how to record their food intake for 2 weekdays and 1 weekend day before and after the intervention. These data have been analyzed with the use of Nutrition 4 software.

### 2.9 Physical activity assessments

The intervention program included recommendation to increase activity as much as 45–60 min' moderate activity three times a week. The shortened form of the international physical activity questionnaire (IPAQ) was used to estimate daily activity before and after the intervention. It includes seven questions related to physical activity associated with work, homework, and leisure time, during the past 7 days. The total metabolic equivalent of task (hours per week) was calculated. The questionnaire had previously been validated in Iran (Mostafai et al., 2018).

### 2.10 Subject compliance

To ensure the subject's compliance, the dietary intakes were assessed using three dietary diary records. Furthermore, to measure adherence to diet protocol, they were asked to attend the laboratory every 2 weeks. Their behavior problems regarding their weight‐loss program were discussed. Subjects had access to one‐to‐one telephone support from the consultants.

### 2.11 Statistical analysis

The software SPSS 20.0 (SPSS Inc) was used to analyze data. Baseline data have been descriptively summarized. We examined the normality of data by Kolmogorov–Smirnov. In the case of data with normal distribution, parametric test was used, whereas in contrast, for the data that are not normally distributed, nonparametric test was used. Within‐group differences were assessed with the use of Wilcoxon. The efficacy of the intervention on the outcomes (between‐group differences) was analyzed via Kruskal–Wallis test. In addition, although low‐calorie diet was similar for all groups, considering that there might be some differences in the following up of the diet between groups, we analyzed the main outcomes. Based on previous studies, the power of the study was calculated, 35 subjects per group (Forssten et al., 2013). To account for an expected 5% dropout rate, 140 participants were recruited.

## 3 RESULTS

### 3.1 Demographic data

Demographic data are shown in Table 1. There were no statistically significant differences at the baseline data including age, gender, duration of sun exposure, education level, and marital status.

**TABLE 1:** Baseline characteristics of groups

	Variable/yogurt	Group 1 (*n* = 31)	Group 2 (*n* = 28)	Group 3 (*n* = 30)	Group 4 (*n* = 30)	*p*‐value
Gender	Male	6 (19.4)	8 (28.6)	9 (30)	11 (36.7)	.516^a^
	Female	25 (80.6)	20 (71.4)	21 (70)	19 (63.3)	
Marital status	Single	23 (26.7)	20 (23.3)	19 (22.1)	24 (27.9)	.472^a^
	Married	8 (24.2)	8 (24.2)	11 (33.3)	6 (18.2)	
Education	Under diploma	23 (24)	23 (24)	25 (26)	25 (26)	.766^a^
	Diploma	8 (34.8)	5 (21.7)	5 (21.7)	5 (21.7)	
Vitamin D status	Sever deficiency	2 (13.3)	3 (20)	8 (53.3)	2 (13.3)	.243^a^
	Deficiency	16 (29.6)	11 (20.4)	12 (22.2)	15 (27.8)	
	Sufficient	12 (24)	11 (22)	13 (26)	14 (28)	
Age		35.37 ± 11.69	40.9 ± 6.75	48.36 ± 9.70	36.35 ± 21.10	.902^b^

Note

Values are expressed as mean ± *SD* or number (percent).

Group 1: RY yogurt, Group 2: PY, Group 3: vitamin D‐fortified yogurt, Group 4: probiotic and vitamin D co‐fortified yogurt.

Abbreviation: MET, metabolic equivalent task‐hours/day.

^a^ Data are tested by Chi‐square test.

^b^ Data are tested by ANOVA.

### 3.2 Anthropometric factor

At baseline, BMI (*p* = 027) and WC (*p* = 010) showed a significant difference between groups. There was a significant weight reduction in each group without any significant difference between groups. There was a significant decrease of WC, WHR, fat mass, PBF, and HC within all groups. After 10 weeks, the comparison difference between groups was not statistically significant (Table 2). Furthermore, comparison changes between groups regarding all anthropometric were not statistically significant.

**TABLE 2:** Anthropometric factors of participant before and after the intervention

Variable/Yogurt	Group 1 (*n* = 31)		Group 2 (*n* = 28)		Group 3 (*n* = 30)		Group 4 (*n* = 30)		*p*‐V^1^	*p*‐V^2^
	Before	After	Before	After	Before	After	Before	After	Before	After
BF^c^(kg)	36.57 ± 6.04	35.19 ± 7.73^a^	38.52 ± 6.04	37.23 ± 6.91^a^	35.05 ± 6.09	34.39 ± 6.90 ^a^	34.08 ± 5.53	32.85 ± 5.39 ^a^	.052	.116
PBF(%)	41 (38.5–42.8)	40 (37.4–42.2)^b^	40.2 (36.3–42.7)	40.1 (36–42.4)^a^	40.6 (37.4–42.8)	39.4 (36.9–41.5)^a^	38.6 (33.7–42.7)	37.9 (32.1–41.7)^a^	.329	.472
TBW^c^(kg)	39.2 ± 5	39.1 ± 4.9	42.3 ± 8.2	41.6 ± 8.1	38.5 ± 7.4	38.9 ± 6.8	40.1 ± 6.9	39.7 ± 7	.390	.451
WHR	0.9 (0.9–0.9)	0.9 (0.9–0.9)	0.9 (0.9–0.9)	0.9 (0.8–0.9)	0. (0.9–1)	0.93 (0.8–1)	0.94 (0.9–0.98)	0.92 (0.90–0.98)	.832	.744
BMI (kg/m^2^)	34.6 (31.9–38)	33.6 (30.7–37.3)^a^	36.3 (33.5–39.5)	35.7 (32.2–38.2)^a^	34.9 (31.9–35.9)	33.8 (31.3–35.7)^a^	33.8 (31.3–35)	33.2 (30.8–34.5)^a^	.027	.059
LBM (kg)	54.1 (49.2–60)	54.2 (49.3–58.5)	54.5 (50.8–65)	55 (49.7–63.8)^a^	49.5 (47.2–60.5)	50.2 (47.1–60.4)	55 (49.5–61.7)	54.7 (48–61.6)	.620	.440
Weight (kg)	90.2 (83.7–97.5)	90.2 (80.9–97.5)^a^	93.6 (87.7–106.8)	91.1 (83–107.8)^a^	87.3 (79.5–97.7)	86.1 (78.5–94.1)^a^	89.7 (84–95.5)	88 (81.1–95.1)^a^	.123	.377
WC (m)	106.7 (102.6–112.2)	99.5 (95–106.5)^a^	109.5 (104.6–115.3)	101 (97–110.5)^a^	103.2 (98.2–111.2)	97.5 (88.3–110)^a^	103.7 (100.3–107.7)	98.5 (93–103)^a^	.010	.081
HC (m)	112.5 (107.8–122.7)	110.5 (105.5–119)^a^	117.2 (111.3–125.8)	113 (107.−120)^a^	115 (106.5–118.6)	109.7 (105.8–115)^a^	111.75 (108.2–116.3)	109 (106.−115.5)^a^	.221	.671

Note

Skewed distributed value are expressed with median (quartile1‐quartile3).

Group 1: RY yogurt, Group 2: PY, Group 3: vitamin D‐fortified yogurt, Group 4: probiotic and vitamin D co‐fortified yogurt.

*p*‐V^1^: *p*‐value stands for significance of between‐group differences before the intervention (Kruskal–Wallis test).

*p*‐V^2^: *p*‐value stands for significance of between‐group differences after the intervention (Kruskal–Wallis test).

Abbreviations: BF, body fat; BMI, body mass index; HC, hip circumference; LBM, lean body mass; PFB, percent body fat; TBW, total body water; WC, waist circumference.

^a^ *p*‐value of <.05.

^b^ *p*‐value of <.001.

^c^ Normal distributed value are expressed as mean ± *SD*.

### 3.3 Calorie and macronutrient intake

Dietary energy and macronutrient intake are shown in Table 3. According to the 3‐day food records, all groups reported a significant reduction in energy intake, protein, fat, and carbohydrate intake, whereas on the contrary, the difference between groups was not significant. Only fat intake in the RY yogurt group did not reduce significantly. Regarding vitamin D, change was not significant either between or within groups. In addition, after adjusting for the protein intake and GLP‐1 as confounders, the result became significant, but it remained nonsignificant between groups. Moreover, comparison changes between groups did not show any significant difference.

**TABLE 3:** Physical activity, sun exposure, energy, and macro nutrient and dietary intake of vitamin D in groups before and after the intervention

Variable/Yogurt	Group 1 (*n* = 31)		Group 2 (*n* = 28)		Group 3 (*n* = 30)		Group 4 (*n* = 30)		*p*‐V^1^	*p*‐V^2^
	Before	After	Before	After	Before	After	Before	After	Before	After
MET (min/day)	0 (0–360)	0 (0–1021.38)	384 (0–1668)	1440 (523–2394)	408 (0–970)	608 (0–1680)	399 (0–960)	372 (0–2274)	.168	.058
Sun exposure (min/day)	30 (10–60)	25 (10–30)	30 (10–60)	15 (7.50–30)	60 (13.75–97.50)	25 (15–60)	30 (10–105)	22.50 (10–42.50)	.649	.549
Energy (kcal)	1863 (1745–2423)	1551 (1395–1757)^b^	2220 (1912–2754)	1820 (1266–2189)^a^	2048 (1648–2759)	1630 (1293–1928)^b^	2799 (2109–3096)	1867 (1392–2732)^b^	.595	.430
Protein (g/day)	75.89 ± 32.06^c^	61.06 ± 17.04^a^ ^,^ ^c^	109.86 ± 80.68^c^	71.86 ± 27.98^a^ ^,^ ^c^	92.17 ± 55.3^c^	75.60 ± 50.69^b^ ^,^ ^c^	102 ± 33.55 ^c^	77.84 ± 33.89^b^ ^,^ ^c^	.595	.012
Carbohydrate (g/day)	297.21 ± 93.81^c^	259.49 ± 122.59^a^ ^,^ ^c^	340.09 ± 181.09^c^	262.61 ± 189.27^b^ ^,^ ^c^	340.09 ± 181.09^c^	262.61 ± 189.27^b^ ^,^ ^c^	372.22 ± 142.93^c^	286.35 ± 143.54^b^ ^,^ ^c^	.105	.153
Fat (g/day)	67.32 ± 16.30^c^	60.04 ± 13.81^c^	49 ± 40.10^c^	40.84 ± 33.78^a^ ^,^ ^c^	80.51 ± 34^c^	66.91 ± 28.68^a^ ^,^ ^c^	92 ± 22.5 ^c^	71.70 ± 27.85^a^ ^,^ ^c^	.001	.019
Vitamin D (µg/dl)	32.96 ± 11.92^c^	37.26 ± 14.12^c^	34.78 ± 15.64^c^	36.67 ± 15.79^c^	28.46 ± 14.99^c^	38.56 ± 15.44^c^	30.44 ± 13.01^c^	36.01 ± 11.05^c^	.263	.323

Note

Group 1: regular yogurt, Group 2: probiotic yogurt, Group 3: vitamin D‐fortified yogurt, Group 4: probiotic and vitamin D co‐fortified yogurt.

Skewed distributed value are expressed with median (quartile1‐quartile3).

*p*‐V^1^: *p*‐value stands for significance of between‐group differences before the intervention (Kruskal–Wallis test).

*p*‐V^2^: *p*‐value stands for significance of between‐group differences after the intervention (Kruskal–Wallis test).

Abbreviation: MET, metabolic equivalent task.

^a^ *p*‐value of <.05.

^b^ *p*‐value of <.001.

^c^ Normal distributed value are as expressed mean ± *SD*.

### 3.4 Gut peptides

The gut peptides data are shown in Table 4. At baseline, the mean level of ghrelin (*p* = 126) and PYY (*p* = 956) showed no significant differences among four groups, whereas there was a significant difference in the GLP‐1 level at the baseline (*p* = 035). Ghrelin increase was significant neither within nor between groups. Furthermore, the increase in GLP‐1 in groups 2 and 3 and the decrease in this peptide in groups 1 and 4 were not statistically significant. Furthermore, the PYY decrease was nonsignificant in 1, 2, and 3 groups, whereas it showed a significant decrease in group 4.

**TABLE 4:** Satiety profile before and after intervention

Variable/Yogurt	Group 1 (*n* = 31)		Group 2 (*n* = 28)		Group 3 (*n* = 30)		Group 4 (*n* = 30)		*p*‐V^1^	*p*‐V^2^
	Before	After	Before	After	Before	After	Before	After	Before	After
GLP−1 (ng/ml)	10.2 (5.1–40)	11.6 (5.1–24)	5.2 (2.4–23)	21 (14–32)	18 (4.4–84)	22 (12–119)	19 (12–14)	20 (43–27)	.035	.195
PYY (ng/ml)	10 (5.7–8)	11 (23–43)	11 (7.3–16)	8.8 (5.2–28)	9.7 (2.9–55)	6.6 (1.7–74)	12 (5.9–28)	5.2 (0.9–20)^a^	.956	.158
Ghrelin (ng/ml)	0.4 (0.3–0.8)	0.4 (0.3–1.1)	0.4 (0.1–0.8)	0.4 (0.2–0.8)	0.5 (0.3–2.9)	0.5 (0.3–5.2)	0.4 (0.4–1.4)	0.7 (0.3–3)	.126	.171

Note

Group 1: RY yogurt, Group 2: PY, Group 3: vitamin D‐fortified yogurt, Group 4: probiotic and vitamin D co‐fortified yogurt.

Skewed distributed value are expressed with median (quartile1‐quartile3).

*p*‐V^1^: *p*‐value stands for significance of between‐group differences before the intervention (Kruskal–Wallis test).

*p*‐V^2^: *p*‐value stands for significance of between‐group differences after the intervention (Kruskal–Wallis test).

Abbreviations: GLP‐1, glucagon‐like pepitde1; PYY, peptide tyrosine‐tyrosine.

^a^ *p*‐value of <.05.

^b^ *p*‐value of <.001.

The difference in GLP‐1 was re‐compared using ANCOVA with dietary intake of protein, fat, BMI, WC, and GLP‐1 as confounder. In this case, the difference in GLP‐1 (*p* = 002) and protein intake (*p* = 02) disappeared.

Moreover, comparison changes between groups showed a significant difference just for GLP‐1 (*p* = 023). Pairwise comparison of GLP‐1 changes was significant just for group 1‐group 2 (*p* = 037) and group 1‐group 4 (*p* = 004).

### 3.5 Physical activity

Regarding physical activity, no difference was seen among the groups before and after the intervention.

### 3.6 Glycemic indices

Data analysis showed that fasting plasma glucose decreased significantly in group 2 (*p* = 008) over 10‐weeks, but it was not significant compared with others (Table 5). Furthermore, after controlling the confounding effects of FBS, it became significant (*p* = 0). However, there was no significant difference between groups. Although comparison changes between groups were not significant for insulin, the difference between groups regarding FBS changes was significant (*p* = 003).

**TABLE 5:** Fasting blood sugar, insulin, and HOMA‐IR before and after intervention

Variable/Yogurt	Group 1 (*n* = 31)		Group 2 (*n* = 28)		Group 3 (*n* = 30)		Group 4 (*n* = 30)		*p*‐V^1^	*p*‐V^2^
	Before	After	Before	After	Before	After	Before	After	Before	After
FBS (mg/L)	92 (85–116)	94 (85–104)	114 (102–119)	93 (89–102)	90 (87–108)	93 (86–98.5)	96 (89.25–104)	95 (89.50–102)	.031	.938
Insulin (µU/ml)	66.56 ± 38.88	14 ± 12.74 *	15.09 ± 10.54	15.01 ± 10.80	15.77 ± 15.45	16.4 ± 14.48	7.04 ± 5.22	2.57 ± 0.97	.059	.057
HOMA‐IR	12.41 ± 6.42	3.28 2.69	1.90 ± 1.15	3.2 ± 1.75	0.37 ± 0.07	0.35 ± 0.08	0.41 ± 0.06	0.41 ± 0.05	.036	.013
QUICKI	0.35 ± 0.08	0.37 ± 0.08	0.37 ± 0.05	0.38 ± 0.06	0.37 ± 0.07	0.35 ± 0.08	0.41 ± 0.06	0.41 ± 0.05	.13	.50

Note

Group 1: regular yogurt, Group 2: probiotic yogurt, Group 3: vitamin D‐fortified yogurt, Group 4: probiotic and vitamin D co‐fortified yogurt.

Skewed distributed value are expressed with median (quartile1‐quartile3).

*p*‐V^1^ *p*‐value stands for significance of between‐group differences before the intervention (Kruskal–Wallis test).

*p*‐V^2^ *p*‐value stands for significance of between‐group differences after the intervention (Kruskal–Wallis test).

Abbreviations: FBS, fasting blood sugar; HOMA‐IR, homeostatic model of insulin resistance; QUICKI, quantitative insulin sensitivity check index.

^*^ *p*‐value of <.05

Insulin resistance did not change in all groups. The difference between groups was significant before (*p* = 036) and after (*p* = 013) the intervention. Comparison changes of HOMA‐IR between groups were nonsignificant. After adjusting HOMA‐IR and FBS as confounders, the result remained nonsignificant Table 5.

Insulin sensitivity increase was nonsignificant either within or between groups. Comparison changes between groups did not show any significant difference.

### 3.7 Effect size

Concerning weight, there was no difference between groups' effect sizes (*p* = 891). Regarding gut peptides, the difference between the effect size of interventions was significant for both anorectic peptides (PYY [*p* = 04] and GLP‐1 [*p* = 003]). Pairwise comparison of GLP‐1 showed that there was a significant difference between ES of RY vs. probiotic and vitamin D‐co‐fortified yogurts (*p* = 048), RY vs. PYs (*p* = 001), and RY vs. Vitamin D yogurt (*p* = 003). The effect size of probiotic was the largest and after that, vitamin D‐fortified yogurt, vitamin D, and probiotic co‐fortified yogurt, and RY yogurt. There was a significant difference just between ES of RY vs. probiotic and vitamin D‐co‐fortified (*p* = 008).

Regarding ghrelin, yogurt fortified with both probiotic and vitamin D had the largest effect size. However, there was no significant difference between groups (*p* = 742).

Furthermore, the effect size of intervention on FBS was significantly different between groups (*p* = 045). There was a significant difference in RY vs. PY (*p* = 014), probiotic vs. vitamin D‐fortified yogurt (*p* = 034), and probiotic vs. probiotic and vitamin D‐fortified yogurt (*p* = 016). The largest effect size was related to PY and after that, RY, probiotic and vitamin D‐fortified yogurt, and vitamin D‐fortified yogurt.

There was a significant difference between groups regarding the effect size of four yogurts on the serum level of vitamin D (*p* = 015). Pairwise comparison showed that there was a significant difference in RY vs. vitamin D‐fortified yogurt (*p* = 018) and probiotic vs. vitamin D‐fortified yogurt (*p* = 002). Vitamin D‐fortified yogurt had the largest effect on the serum level of vitamin D, and after that, probiotic plus vitamin D, RY, and PY (Table 6).

**TABLE 6:** Effect size of intervention on main outcome

Variable/Yogurt	Group 1 (*n* = 31)	Group 2 (*n* = 28)	Group 3 (*n* = 30)	Group 4 (*n* = 30)	*p*‐V
Weight ES	1 (−0.15–3.02)	1.5 (0.07 ± 4.75)	1.20 (−0.45 ± 3.20)	1.20 (0.02 ± 2.90)	.891
GLP−1 ES	0.50 (−1.3 ± 2.40)	−14.2 (−21 ± 4.30)	−0.80 (−13.95 ± 3.35)	0.70 (−0.55 ± 11.55)	.003
PYY ES	−0.05 (−3.57 ± 2.32)	2 (−2.40 ± 9.65)	−0.1 (−3.35 ± 9.50)	2.90 (−0.75 ± 13.40)	.040
Ghrelin ES	0 (−0.20 ± 0.20)	0 (−0.1 ± 0.12)	0 (−0.20 ± 0.15)	0 (−0.05 ± 0.10)	.742
Vitamin D ES	0 (0 ± 0.25)	0 (0 ± 0)	0 (0 ± 1)	0 (0 ± 1)	.015
FBS ES	−4 (−6 ± 5.50)	−14 (−31 ± −9)	−2 (−15 ± 4.5)	−4 (−8.75 ± 7.25)	.045
HOMA‐IR ES	0 (−4.52 ± 0.26)	0 (−0.27 ± 0.46)	0 (0 ± 0.76)	0 (−0.05 ± 0.08)	.562

Note

Group 1: regular yogurt, Group 2: probiotic yogurt, Group 3: vitamin D fortified‐ yogurt, Group 4: probiotic and vitamin d co‐fortified yogurt.

*p*‐V stands for significance of between‐group differences (Kruskal–Wallis test).

Abbreviations: FBS EF, fasting blood sugar effect size; ghrelin ES, ghrelin effect size; GLP‐1 ES, GLP‐1 effect size; HOMA‐IR ES, HOMA‐IR effect size; PYY ES, PYY effect size; Vitamin D ES, vitamin D effect size; Weight ES, weight effect size

## 4 DISCUSSION

In the present 10‐week intervention, the daily intake of probiotic‐fortified yogurt or vitamin D‐fortified yogurt during a 10‐week calorie restriction increased the anorectic peptides, GLP‐1, without any change in ghrelin. Since anorectic gut hormones may play a key role in promoting the sustained weight loss and satiety feeling, whereas the increase in ghrelin may contribute to the lack of sustained weight loss in low‐calorie diet, these observations confirm that the recent recommendation made in many guidelines to consume a healthy diet containing probiotic product and suppling vitamin D sufficiently is likely to improve microbiota–gut–brain axis and as a result adjust the hemostasis and daily intake. There are studies that show that higher fasting GLP‐1 is associated with higher resting energy expenditure and fat oxidation (Pannacciulli et al., 2006).

Surprisingly, in spite of our presumption, consumption of vitamin D and probiotic‐co‐fortified yogurt decreased PYY significantly. This finding raises the possibility that probiotic and vitamin D may impose a negative effect on each other.

The modulatory effect of probiotics on GLP‐1 seen in our study is in accordance with other studies where it was identified as probiotic supplementation which can increase these anorectic hormones level (Falcinelli et al., 2016; Yadav et al., 2013). There are several potential mechanisms for the effect of probiotics on satiety, and on top of that, affecting dysbiosis of the gut flora. Emerging evidence has shown that the function of gut microbiota affects not only intestinal physiology but also microbiota–gut–brain axis which is strongly affected (15). Consequently, changes in this axis are associated with changes in hunger and satiety responses. Based on a homeostatic model of appetite regulation which was suggested by Sergueï O. Fetissov et al. (Fetissov, 2016), there is an integration between nutrient‐induced dynamics of gut bacterial growth and activation of host intestinal satiety signal by nutrients.

SCFASs are another metabolite of gut microbiota that participates in long‐term control of appetite. Butyrate, the well‐known metabolite of microbiota, binds to the specific receptors on colonic L‐cell and triggers the secretion of anorectic hormones (29). Based on mice studies, central homeostatic of appetite was affected by the acetate. This SCFA suppresses appetite by crossing the blood–brain barrier which further acts directly on central neurons which modulate satiety and, therefore, potentially triggers a reduction in food and energy intake.

In contrast to our findings, Hajimohammadi et al. reported that consumption of vitamin D‐fortified drink (without calorie restriction) increased the ghrelin level significantly (Hajimohammadi et al., 2017). The proposed mechanism of the modulatory effect of vitamin D might be related to the function of its receptor VDR (Kong et al., 2008). This effect may occur by the critical effect of VDR on regulating intestinal homeostasis by inhibiting pathogenic bacterial penetration to the host blood circulation, preventing inflammation, and maintaining cell integrity (Yoon & Sun, 2011). Furthermore, it has been indicated in an experimental study that VDR affects the expression of genes in some parts of the stomach and as a result, regulates gastric hormone secretion (Stumpf, 2008). Vitamin D deficiency might deteriorate energy hemostasis by augmenting the existing dysbiosis burden on obese people. On the other hand, it has been indicated by accumulating evidence that vitamin D plays a critical role in insulin secretion (Bland, 2004). Based on mice studies, damaged VDR impaired insulin secretion (Yoon & Sun, 2011). It can be postulated that vitamin D regulates insulin secretion via VDR. Therefore, the second possible mechanism of vitamin D action might be related to settling insulin resistance problem in obese people, thus promoting reversing of the impaired glp‐1 and ghrelin responses (Verdich et al., 2001).

Concerning physical activity, although all participants were encouraged to be active, it did not make any significant effect on results. A plethora of studies have examined the appetite‐related responses after different types of exercise. Appetite perceptions typically return to resting control values within 30–60 min of exercise cessation. Indeed, energy deficits induced by exercise are short term and do not lead to acute compensatory responses in appetite, energy intake, or gut peptides (Douglas et al., 2017).

Regarding the effect of diet on serum level of GLP‐1 and PYY, there are conflicting results (Lean & Malkova, 2016; O’Connor et al., 2016). Our result is inconsistent with previous research demonstrating the effect of negative energy balance on gut peptides (Pasiakos et al., 2012; Sumithran et al., 2011). We find no significant difference between the effect size of low‐calorie diet and physical activity on all groups and, as a result, weight loss. Thus, the changes in gut peptides might be related to the yogurts. Apart from the daily intake of yogurts, there were some differences in the design of studies, for instance, the duration of the intervention and the time in which measurement was done. On the other hand, the problem with other previous studies is that they did not take into account the confounding effect of changing diet composition and physical activity in the weight‐maintenance phase. To address this gap, in our study, measurement was taken just after the intervention without any weight‐maintenance phase.

Changes in gut peptides in RY can be partially explained by the beneficial effect of components such as protein, calcium, yogurt culture on increasing satiety feeling, which has been supported by several studies and a recent RCT meta‐analysis (7).

Regarding glycemic indexes, similar to the result from other clinical trials, our study showed that daily consumption of probiotic‐fortified yogurt decreases the FBS level significantly compared with other types of yogurts (Ivey et al., 2015; Rezaei et al., 2017). However, our result points out that an increase in the daily intake of probiotic and vitamin D‐fortified yogurts along with increasing physical activity during energy restriction does not impose any beneficial effect on insulin sensitivity and insulin resistance improvement during weight loss.

The strength of the present study is being the first randomized controlled trial that assessed the synergist effect of probiotic and vitamin D in the yogurt matrix in a subject undergoing low‐calorie diet. The second one is that, unlike previous studies, measurement was taken in a negative energy balance period. At the top of the mentioned reasons, the benefits which have been shown are in spite of dynamic effects arising from an acute negative energy balance.

The limitation inherent in the use of self‐reported diet and physical activities is admitted. Even though many precautions were taken to optimize compliance (regular visits to the laboratory and discussion with the dietician, return of yogurt bowels, empty or not), it is impossible to ascertain the level of compliance, due to lack of an adequate biomarker.

## 5 CONCLUSION

Daily intake of 100 g PY or vitamin D‐fortified yogurt in healthy subjects undergoing a low‐calorie diet over 10 weeks improved the anorectic hormone, GLP‐1 without any change in ghrelin. This result suggests a promising approach for controlling the negative effect of negative energy balance during diet therapy on gut hormones.

## CONFLICT OF INTEREST

The authors declare no conflict of interest in this study.

## Data Availability

The data sets used and/or analyzed during this study are available from the corresponding author on reasonable request. Permission for use was received by the ethics committee of medical university of Kermanshah.
